# Factors influencing the pigment composition and dynamics of photoautotrophic picoplankton in shallow eutrophic lakes

**DOI:** 10.1371/journal.pone.0267133

**Published:** 2022-05-26

**Authors:** Marju Tamm, Tiina Nõges, Peeter Nõges, Kristel Panksep, Priit Zingel, Helen Agasild, Rene Freiberg, Triin Hunt, Ilmar Tõnno

**Affiliations:** Chair of Hydrobiology and Fishery, Institute of Agricultural and Environmental Sciences, Estonian University of Life Sciences, Tartu, Estonia; INRA/Sorbonne University, FRANCE

## Abstract

Photoautotrophic picoplankton (0.2–2 μm) can be a major contributor to primary production and play a significant part in the ecosystem carbon flow. However, the understanding about the dynamics of both eukaryotic and prokaryotic components of picoplankton in shallow eutrophic freshwater environments is still poor. Very few studies in these ecosystems reveal the taxonomic composition of picoeukaryotes. The main objective of this study was to investigate the seasonal dynamics of phototrophic picoplankton with the emphasis on the eukaryote community composition in a large shallow, eutrophic lake of the northern temperate zone (Lake Võrtsjärv). Phytoplankton pigments were employed to determine the taxonomic composition of photoautotrophic picoplankton. We found out that photoautotrophic picoplankton constitutes an important part of the phytoplankton community in Lake Võrtsjärv and its contribution can be highly variable (from ~9.3% to ~39%) in different years. The eukaryotic photoautotrophic picoplankton was dominated by diatoms followed by chrysophytes and other minor groups. Picoeukaryotes were prevailing in low-light conditions and low temperatures as their predominance in the picoplankton community was tightly linked to the presence or absence of ice cover. Ice cover strongly suppressed the growth of picocyanobacteria. Total phosphorus, turbidity and metazooplankton abundance had a clear relationship with photoautotrophic picoplankton chlorophyll *a*.

## Introduction

Photoautotrophic picoplankton (PPP) represents the smallest (0.2 to 2 μm) photoautotrophic organisms present in aquatic environments [1]. PPP is ubiquitous in a large range of freshwater systems from deep ultra-oligotrophic to shallow eutrophic lakes, significantly contributing to ecosystem carbon flow [2–4]. Most of the World Ocean is also dominated by the PPP and the contribution of PPP to the total primary production in oligotrophic lakes and oceans may reach up to 50–90% [5–8]. Consequently, PPP provides a significant source of energy and carbon in aquatic food webs playing an essential role in the microbial loop [9].

PPP is commonly divided into two large segments—picoprokaryotes and picoeukaryotes (Peuk). Previous studies indicate that the role of picoprokaryotes i.e. picocyanobacteria (Pcy) relative to the total phytoplankton tends to be more significant in the oligotrophic lakes while the overall abundance of Pcy might be higher in the eutrophic lakes [9–11]. Currently it is considered as a general rule for the PPP that the highest biomass occurs in the most productive lakes and the lowest biomass in the lakes with poor production. However, the relative proportion of PPP to the total phytoplankton biomass is the smallest in the most productive lakes and vice versa [12].

Picoeukaryotes are generally considered to be less abundant than picocyanobacteria, Peuk are rather important in the acidic dystrophic, eutrophic and humic lakes [9, 13–16]. Despite their lower abundance it is acknowledged that Peuk are major contributors to phytoplankton biomass [17] and better competitors at lower light and temperature levels and thus better fit to grow under the ice cover [18]. Also, Peuk are recognized to control bacterioplankton population impacting the energy and nutrient flows in microbial food webs [19] and thus being significant players in aquatic ecosystems. Previous research has established that the proportion of picoeukaryotes and -prokaryotes to total phytoplankton biomass may vary largely among lakes with similar limnological properties and also within the seasonal cycle [3, 18, 20]. Several authors have pointed out that chlorophyll *a* (Chl *a*) might be rather poor predictor for PPP number and the linear relationship between the trophic state and PPP abundance is debatable especially in shallow lakes and turbid water bodies [15, 20–22].

Owing to methodological issues, picocyanobacteria have been in the focus of research for a much longer time than picoeukaryotes. Peuk have been treated as a bulk assemblage for decades since most of the traditional approaches to PPP quantification (epifluorescence microscopy, flow cytometry) are deficient in taxonomic resolution [23]. More recently, molecular methods have exposed a wide diversity of Peuk and have started to fill knowledge gaps in their taxonomic diversity [24–26]. However, metabarcoding as a qualitative analysis does not provide data for the comparisons of the relative abundances among different taxonomic groups [27, 28]. Pigment-based chemotaxonomy is therefore a great tool to fill the knowledge gap in the taxonomic diversity of picoeukaryotes [29, 30]. As a considerable amount of marker pigments is available for chemotaxonomic analysis, pairing them with interpretation tools (e.g. Chemtax model) allows partitioning of the Chl *a* biomass among different phytoplankton groups [31].

Whereas the interest in the role of Peuk has grown during the past decades, the majority of studies have still focused on marine environments [32, 33] and fewer studies depict both taxonomic composition and seasonal dynamics of Peuk in different types of freshwater systems [3, 4]. Most of the studies have been carried out in deeper oligotrophic lake systems while only some research has been done in shallow eutrophic freshwater environments [16, 20]. Very few of them reveal the taxonomic composition of Peuk [4, 34]. Moreover, as the relative importance of PPP is not necessarily associated with the trophic state of shallow lakes, it has been suggested that PPP needs more attention in the hypertrophic end of the trophic spectrum to clarify its success in these conditions [20]. Hence, this paper targets the seasonal dynamics of different PPP components with the emphasis on the Peuk community composition in a large shallow, eutrophic temperate lake. Additionally, this study gives an insight to the Peuk community dynamics during ice-cover when the growth of all phytoplankton is mainly limited by light.

The objectives of this study were to: (1) describe the taxonomic groups of Peuk via pigment-based chemotaxonomy on the seasonal scale, (2) examine the temporal variation of pro- and eukaryotic PPP and their share in the total phytoplankton biomass and (3) investigate the relationship between abiotic and biotic environmental parameters, and Peuk, Pcy dynamics in a large and shallow eutrophic lake.

## Materials and methods

### Study site

Our survey was carried out in a large (270 km^2^) and shallow (mean depth of 2.8 m, maximum 6 m) polymictic Lake Võrtsjärv (58◦ 15′ 7′′ N and 26◦ 1′ 47′′ E), situated in central-southern Estonia. Võrtsjärv is turbid with the average Secchi depth around 1 m during the ice-free vegetation period. Lake is characterized as highly eutrophic—mean total phosphorus (TP) 0.045 mg L^-1^, mean total nitrogen (TN) 1.3 mg L^-1^ and annual gross primary production 208 g C m^−2^ [35]. Location in the hemiboreal zone makes Võrtsjärv ice-covered on average 135 days per year [36]. Võrtsjärv is strongly affected by the climatic factors—directly by air temperature and indirectly through the fluctuations of non-regulated water level that depends on the amount of precipitation [37].

Primary production in the lake is predominantly performed by cyanobacteria (94%) according data of Cremona et al. [38]. The two most abundant cyanobacterial species are non-N_2_-fixing slow-growing and highly shade tolerant *Limnothrix planctonica* (Wolosz.) Meffert and *L*. *redekei* (Van Goor). These species are followed by other less numerous filaments e.g. *Planktolyngbya limnetica* (Lemm.) Kom.- Legn.; *Aphanizomenon skujae* Kom.- Legn. and Cronb [39]. Diatoms are the next more abundant group, mainly dominated by the genera *Aulacoseira* or *Synedra* followed by other minor phytoplankton groups i.e. chlorophytes, cryptophytes, chrysophytes and dinoflagellates [40].

### Sampling

Depth-integrated water samples were collected roughly once a month in 2014 (n = 10) and 2018 (n = 14, with one sample collected in January 2019) from the monitoring station near the lake’s deepest point. Water was collected with a Ruttner sampler (1.5 L) with 0.5 m increments starting from ~0.5 m below water surface and finishing half meter above the sediment surface. Samples were mixed in a 30-L barrel and further processed onshore a few hours later. Subsampling of the depth-integrated water for water chemistry, high-performance liquid chromatography (HPLC) and microscopy was done in the laboratory. Additionally, Secchi depth, water temperature and general meteorological conditions were recorded in the field. No specific permits were required for any part of the study, field studies did not involve any endangered or protected species.

### Pigment extraction and HPLC analysis

For HPLC analysis, 100–700 mL of sampled lake water was gently vacuum filtered (<0.2 bar) through 47-mm Whatman GF/F (0.7 μm pore size) filters, as this type of filters are used in most standard protocols for pigment analysis of total phytoplankton community (e.g. Chavez et al., 1995; Aminot and Rey, 2002). To analyze picoplankton, water was gently vacuum filtered (<0.2 bar) sequentially through the following 47-mm filters with decreasing pore size: 12 μm, 2 μm (both Whatman Nuclepore) and 0.2 μm (Whatman nylon membrane filters). It was ensured that non of the prefilters got clogged during the filtration. If any signs of the filters clogging appeared, we reduced the volume of water being filtered and restarted the filtering. Filters were stored in 5-mL plastic vials, frozen immediately and kept in the darkness at -70 °C prior to analysis. Pigments were extracted in 2 mL 100% acetone with internal standard and sonicated (Branson 1210) for 5 min. After keeping the samples at -20 °C for 24 h, the extracts were filtered through 0.45 μm syringe filters (Millex LCR, Millipore) and stored in dark refrigerated until HPLC analysis (for details see 40).

### Chemtax analysis

Chemtax program (Version 1.95) was used to calculate the abundance (in the units of Chl *a*) of different phytoplankton groups based on the HPLC measurements. Chemtax calculates the relative abundance of phytoplankton classes in reference to total Chl *a* in a water sample employing unique marker pigments and marker pigment: Chl *a* ratios [41]. The diagnostic pigments for the phytoplankton groups identification were chosen according to the microscopy data. In total six algal groups were defined and loaded into the Chemtax program—chlorophytes, dinoflagellates, chrysophytes, diatoms, cryptophytes and cyanobacteria. The pigment ratios and marker pigments used in the current work were derived from Tamm et al. [40] as this study was conducted in Võrtsjärv and the marker pigment: Chl *a* ratios were already validated with microscopy data. During the analysis, 60 ratio matrices were constructed based on the initial ratios, and 10% (n = 6) of the ratios with lowest residual root means square (RMS) were averaged and run until the ratios became stable as proposed by Higgins et al. (2011).

### Microscopy counts

Phytoplankton samples for fraction >2 μm (100 mL) were fixed with 2% acid Lugol’s iodine solution. Approximately 400 counting units (cells, colonies, coenobia, trichomes) were counted and identified to species level under an inverted microscope in accordance with the Utermöhl’s [42] technique. At least one full transect was counted at 400x magnification ranging from one edge of the counting chamber to the other along the chamber’s diameter. Entire chamber area was examined for the less abundant large specimens and colonies. The biovolumes were calculated by applying the formulae of the closest geometric shape [43] and converted into wet biomass assuming a specific gravity of 1 g mL^−1^.

Ciliate samples were fixed with the acidified Lugol’s iodine and Utermöhl [42] technique was used for the determination of ciliate abundance, biomass and community composition. 10–20 mL of water was allowed to settle for at least 24 h in counting chambers. Ciliates were enumerated and identified with an inverted microscope (Nikon Eclipse Ti-S) at 400x magnification. The entire contents of each Utermöhl chamber was surveyed. Abundances were counted in four size classes of <20, 20–40, 40–100 and >100 μm.

For metazooplankton samples, 10 L of the depth-integrated water was filtered through a 48-μm mesh plankton net and fixed with acidified Lugol’s iodine solution (0.5% final concentration). Metazooplankton composition and biomass were analyzed in a Bogorov chamber in triplicate subsamples (4 mL) under a dissecting microscope Nikon AZ100 at 80x magnification. Crustacean length was converted to wet weight as described by Studenikina and Cherepakhina [44], and Balushkina and Winberg [45]. The individual wet weights of rotifers were estimated from average lengths, according to Ruttner-Kolisko [46].

### Statistical analysis

All statistical analyses and figures were performed with the R software [47]. To test the associations between continuous variables, Pearson correlation (r_p_) coefficient was calculated. For the data lacking normal distribution, either square root or logarithmic transformation was applied beforehand. If this did not result in normally distributed data, Spearman rank-order correlation (r_s_) was used. To test if a continuous variable (e.g. PPP, Chl *a*) differed statistically significantly between the two study years, Welch Two Sample t-test was applied. To analyze which environmental parameters explain the variations of PPP-Chl *a*, Peuk and Pcy throughout the years, forward stepwise multiple regressions were performed. The models were built using a mixed stepwise algorithm, that picks a model by using Akaike information criterion (AIC). The effect of extreme values was reduced by square root transforming both the environmental and dependent variables. Variance inflation factor (VIF) was applied for detecting collinearity between the variables using usdm package. The VIF threshold was set at 5 and collinear descriptors were not included in the models.

## Results

### Physical and chemical properties

Water temperature in Lake Võrtsjärv ranged from 0.5 to 24 °C During the vegetation period (May-October) the mean temperature was slightly lower in 2014 (15.6 °C) than in 2018 (18.7 °C). Lowest values occurred in the late autumn and winter (November to March), warmest months were July and August. In 2014 there were two periods of ice cover: from January to March (56 days) and from November to December (17 days). These periods were longer and more consistent in 2018 from January to April (88 days) and from the end of November to January 2019 (more than 60 days).

In 2014 the water level was rather stable ranging only within 0.66 m as the year already started with significantly lower water level than usual. On the contrary, 2018 began with exceptionally high water level that gradually dropped by 1.54 meters.

**Fig 1:**
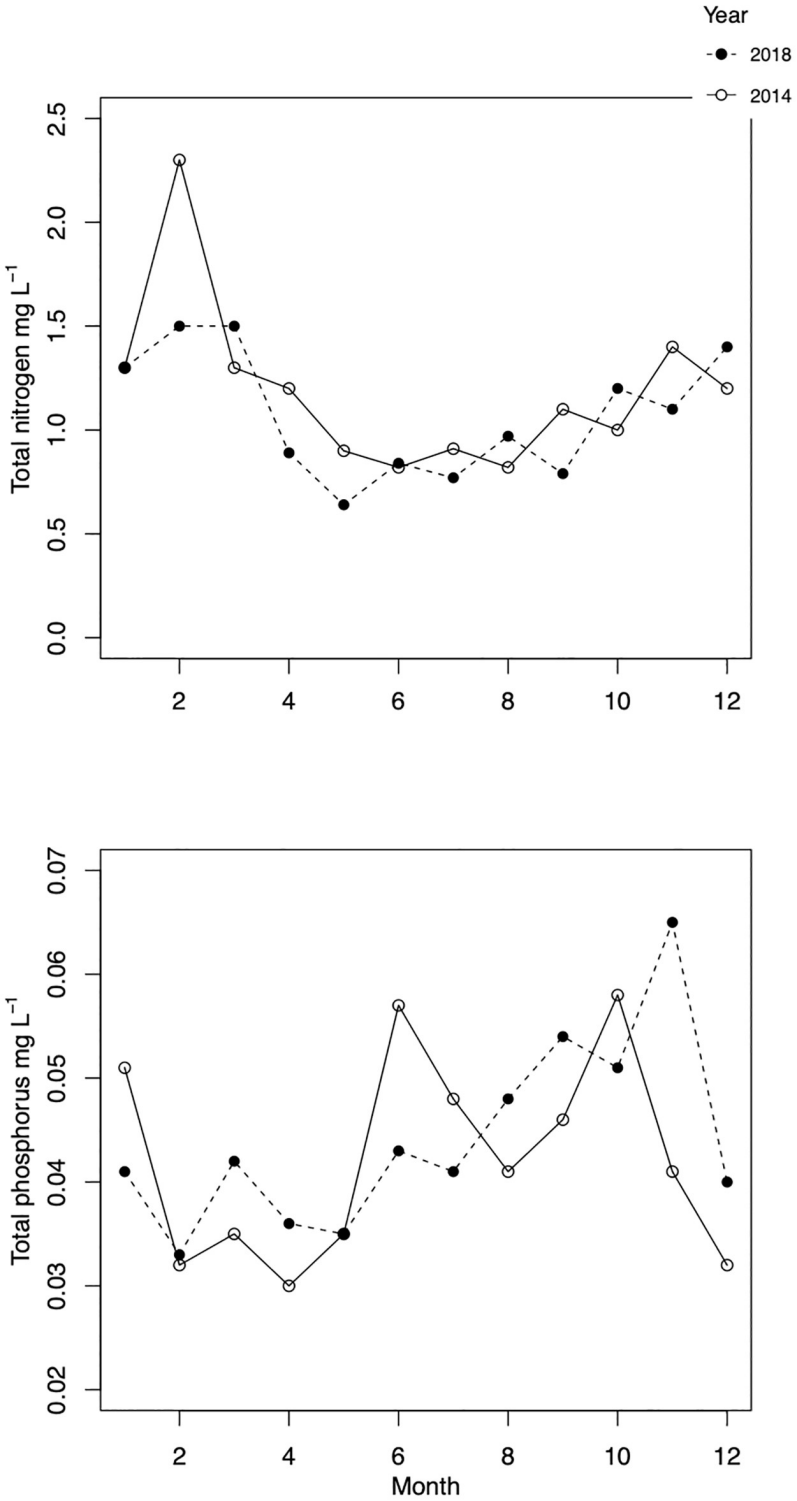
Seasonal dynamics of total phosphorus (TP, mg L^-1^, lower) and nitrogen (TN, mg L^-1^, upper) in 2014 (solid line, empty circles) and 2018 (dashed line, filled circles).

Despite low water level in both years, TP concentrations remained mostly below the long-term average of 0.05 mg L^-1^ and were similar throughout the years (Fig 1, lower, Table 1). TN concentrations were also comparable during the studied years and followed similar seasonal dynamics (Fig 1, upper). TN concentrations dropped during the vegetation period but remained above 0.6 mg L^-1^, highest values occurred from December to March (Fig 1).

**Table 1:** Mean and range of chemical, physical and biological parameters measured in Lake Võrtsjärv in years 2014 and 2018.

Characteristic	2014	2018
Chl *a*, mg m^-3^	21.8 (5.2–32.1)	14.3 (2.2–29.3)
Chl *a* 0.2, mg m^-3^	1.7 (0.6–3.6)	7.6 (0.05–33.3)
Phytoplankton wet biomass g m^-3^	19.2 (2.8–41.8)	22.5 (1.3–41.1)
Ciliates, individuals mL^-1^	92 (25–184)	118 (12–280)
Metazooplankton, individuals L^-1^	1107 (58–3666)	364 (16–953)
NH_4_-N mg L^-1^	0.08 (<0.01–0.12)	0.06 (<0.01–0.12)
NO_2_-N mg L^-1^	0.01 (<0.003–0.022)	0.12 (<0.003–0.012)
NO_3_-N mg L^-1^	0.36 (0.01–1.8)	0.34 (0.02–1)
PO_4_-P mg L^-1^	0.01 (0.007–0.028)	0.01 (0.01–0.024)
TP, mg L^-1^	0.04 (0.03–0.06)	0.04 (0.03–0.07)
TN, mg L^-1^	1.19 (0.82–2.3)	1.05 (0.6–1.5)
Secchi depth, m	0.8 (0.5–1.8)	0.8 (0.4–1.8)
pH	8.4 (8.2–8.7)	8.5 (7.3–8.2)
Temperature, °C	9.2 (0.5–23)	12.3 (0.1–24)

The range of water transparency was very similar during the two years (Table 1) but better light conditions persisted for a longer period in winter-spring of 2018 (February to April).

### Total and picoplankton Chl *a*

**Fig 2:**
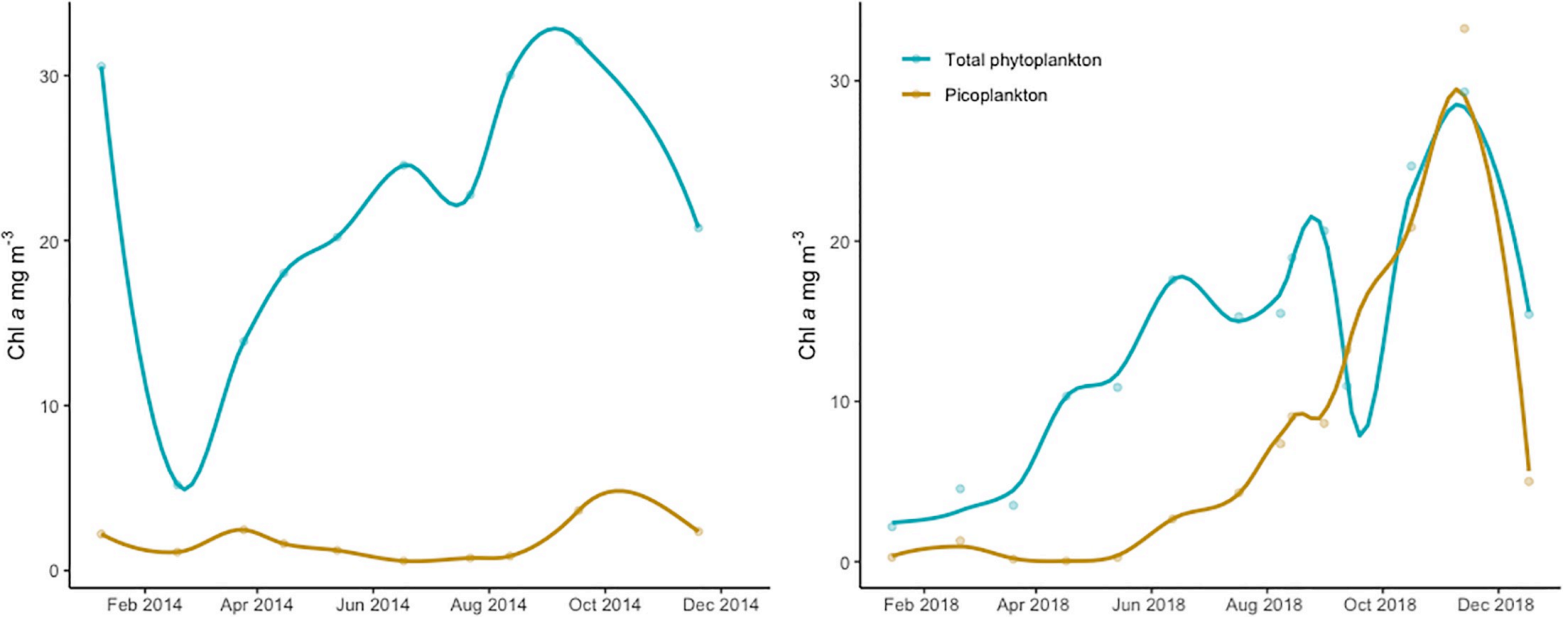
Chl *a* concentrations of total phytoplankton (GF/F, green) and phototrophic picoplankton (0.2–2 μm) size fraction (PPP, yellow) in Lake Võrtsjärv during 2014 (left) and 2018 (right). Loess line is fitted on the data.

Throughout the study total Chl *a* on GF/F filters ranged from 2.18 to 32.09 mg m^-3^ (Fig 2). The mean total Chl *a* value during the vegetation period was significantly (t-test, t = -10.072, df = 4, p<0.01) lower in 2018 (17.4 mg m^-3^) than in 2014 (25.9 mg m^-3^). In both years the phytoplankton wet biomass and Chl *a* started building up in the middle of summer reaching the peak around October-November. Unusually high Chl *a* value occurred in January 2014 (Fig 2, left, 30.6 mg m^-3^). With this exception, lowest Chl *a* values generally appeared from January to March. Phytoplankton >2μm quantified by microscope was usually dominated by filamentous cyanobacteria *Limnothrix planctonica* and *L*. *redekei* with diatom *Aulacoseira* sp. as a subdominant in some months. Other species were represented in very small amounts.

Chl *a* measured from the 0.2 μm filters representing the picoplankton (PPP-Chl *a*) was between 0.05 and 33.27 mg m^-3^ being the lowest in March-April 2018 (0.1 mg m^-3^) and in June-July 2014 (0.67 mg m^-3^). The mean PPP-Chl *a* during the vegetation period was significantly (t = 2.53, df = 4, p< 0.05) higher in 2018 (8.3 mg m^-3^) compared to 2014 (1.4 mg m^-3^). Unfortunately, the data from 2014 is lacking measurements from October and December making the late autumn dynamics a little less apparent. However, it seems that while in 2018 picoplankton was peaking in October-November (Chl *a* 20–33 mg m^-3^), in 2014 the culmination happened slightly earlier—maximum Chl *a* value appeared already in September (3.6 mg m^-3^) and declined by November (2.4 mg m^-3^). The variation of picoplankton Chl *a* between the two years was most substantial in November (Fig 2).

### Relative proportion and taxonomic composition of PPP

**Fig 3:**
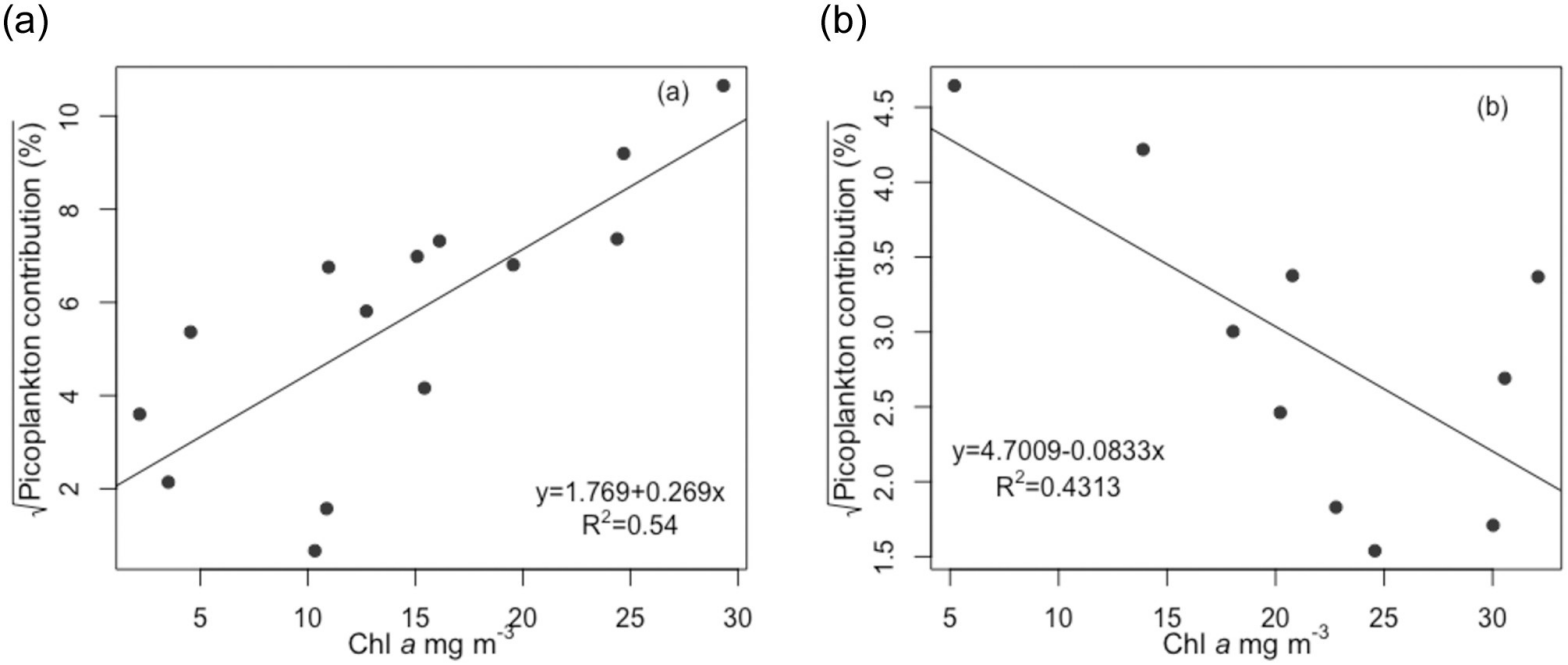
Relationship between total Chl *a* concentration and the proportion of phototrophic picoplankton Chl *a* in 2018 (a) and 2014 (b). Regression line is fitted to data.

The average contribution of PPP to total phytoplankton was significantly higher (t-test, t = 3.36, df = 14.4, p<0.01) in 2018 (~39%) than in 2014 (~9.3%). Throughout the study period, total Chl *a* was negatively correlated (r_p_ = -0.61, p<0.01) with the proportion of picoeukaryotes and positively with the proportion of picocyanobacteria (r_s_ = 0.69, p<0.001). In 2018 a very strong positive correlation (r_p_ = 0.88, p<0.001) was found between total Chl *a* and PPP-Chl *a* concentrations, the higher total Chl *a* values associated with a bigger relative proportion of PPP-Chl *a* (r_p_ = 0.76, p<0.05, Fig 3a). Interestingly, opposite association was observed in 2014 when total Chl *a* concentration was negatively correlated with the proportion of picoplankton Chl *a* (r_p_ = -0.66, p<0.05, Fig 3b).

**Fig 4:**
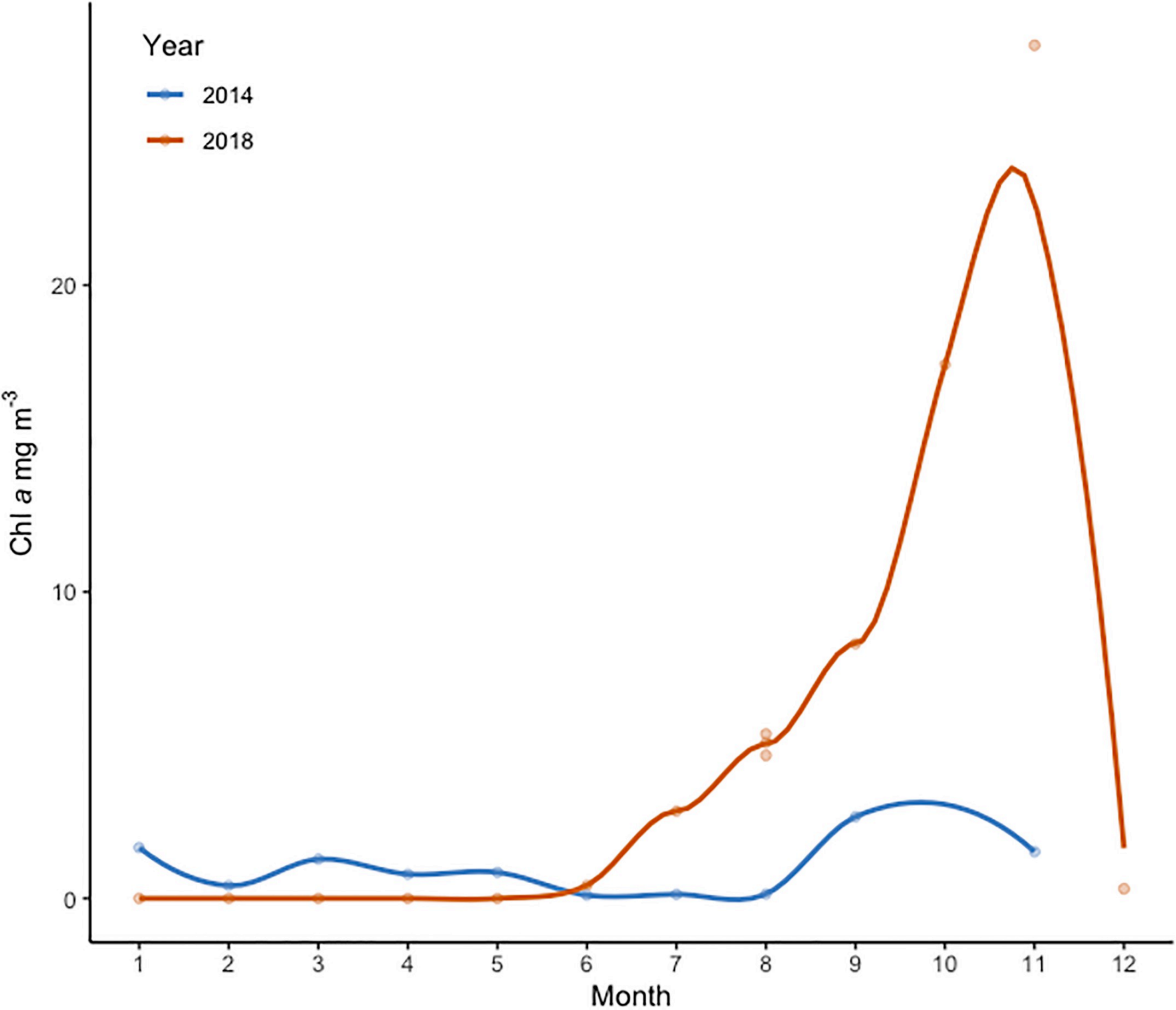
Seasonal dynamics of picocyanobacteria Chl *a* (mg L^-1^) in Lake Võrtsjärv in 2014 (blue) and 2018 (red). Loess line is fitted on the data.

**Fig 5:**
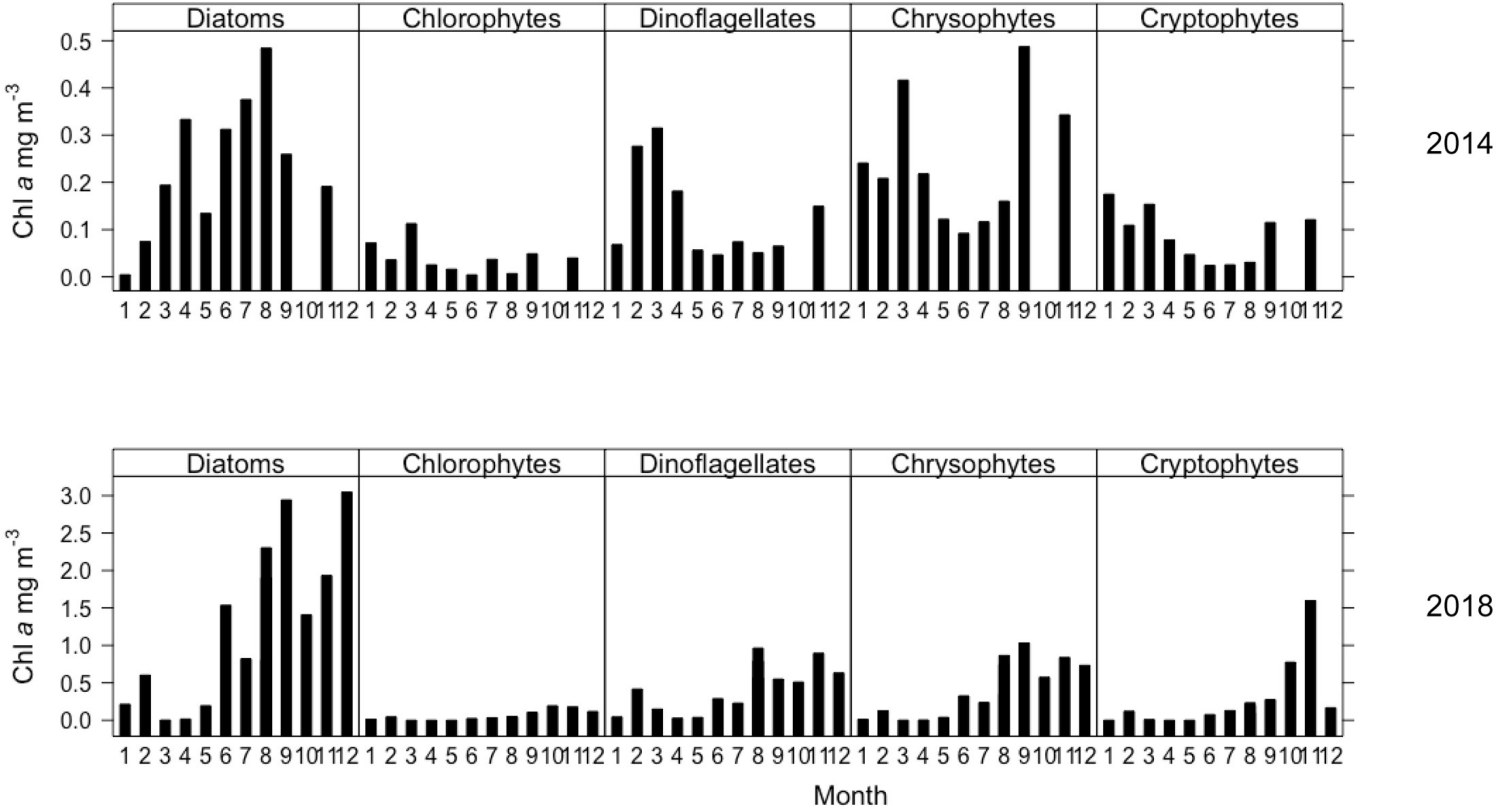
Taxonomic composition of phototrophic picoeukaryotes (0.2–2 μm) in Lake Võrtsjärv in 2014 (upper) and 2018 (lower). Chl *a* concentrations for each group are based on pigment analysis, calculated by Chemtax.

**Fig 6:**
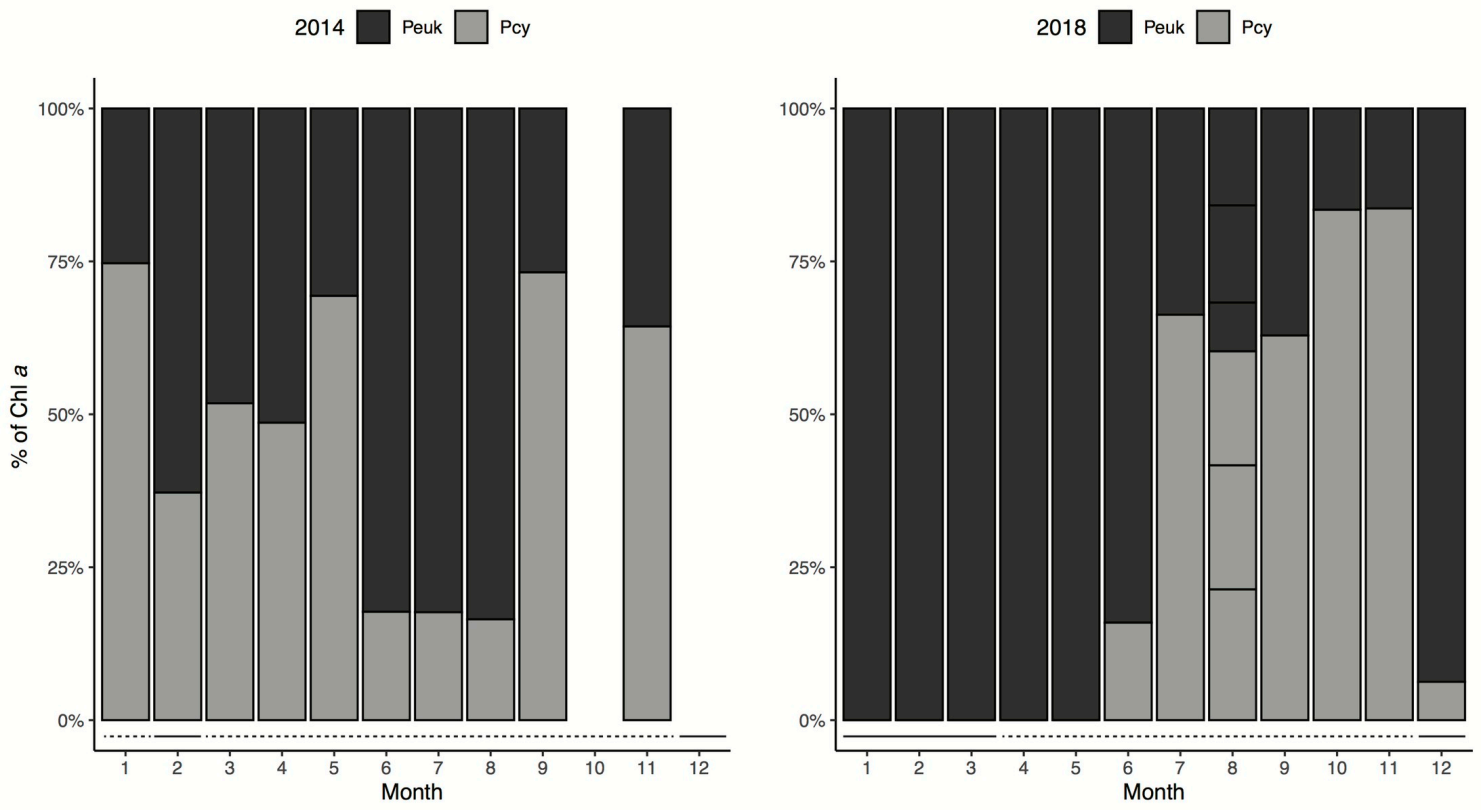
Relative contribution of phototrophic picoeukaryotes and picocyanobacteria in Lake Võrtsjärv in 2014 (left) and 2018 (right). Solid line represents the months where samples were collected from under ice. Dashed line refers to months without ice cover during sampling.

Chemtax analysis indicated that most of the PPP-Chl *a* in Võrtsjärv belonged to cyanobacteria. Peuk was usually dominated by diatoms (Figs 4 and 5), followed by other minor groups (e.g. chrysophytes and dinoflagellates). Both cyanobacteria and diatom Chl *a* biomass started to build up in summer and increased further during the autumn. Cryptophytes and chlorophytes were the least abundant groups, the latter showing very constant Chl *a* biomass during the two years. Cyanobacteria were absent from the PPP community from January to May 2018 (Figs 4 and 6).

The seasonal dynamics of the pro- and eukaryotic component of PPP was very different between the studied years (Fig 6). In 2014 the minima of picocyanobacteria was observed in summer months (June to August) but in 2018 in early spring (March-April). In 2018 small eukaryotes clearly dominated the PPP community in winter-spring and cyanobacteria became more important in late summer and autumn. However, the Chl *a* biomass of Peuk was low in winter-spring and attained the annual maxima from August to December. The Peuk dynamics was rather similar in two years but in 2014 high Chl *a* values were recorded also in winter-spring. In 2014 the Peuk was dominating the PPP community only in February and during summer months (June to August). In all samples collected during ice cover, picoeukaryotes built up the majority of PPP-Chl *a*.

### Zooplankton

**Fig 7:**
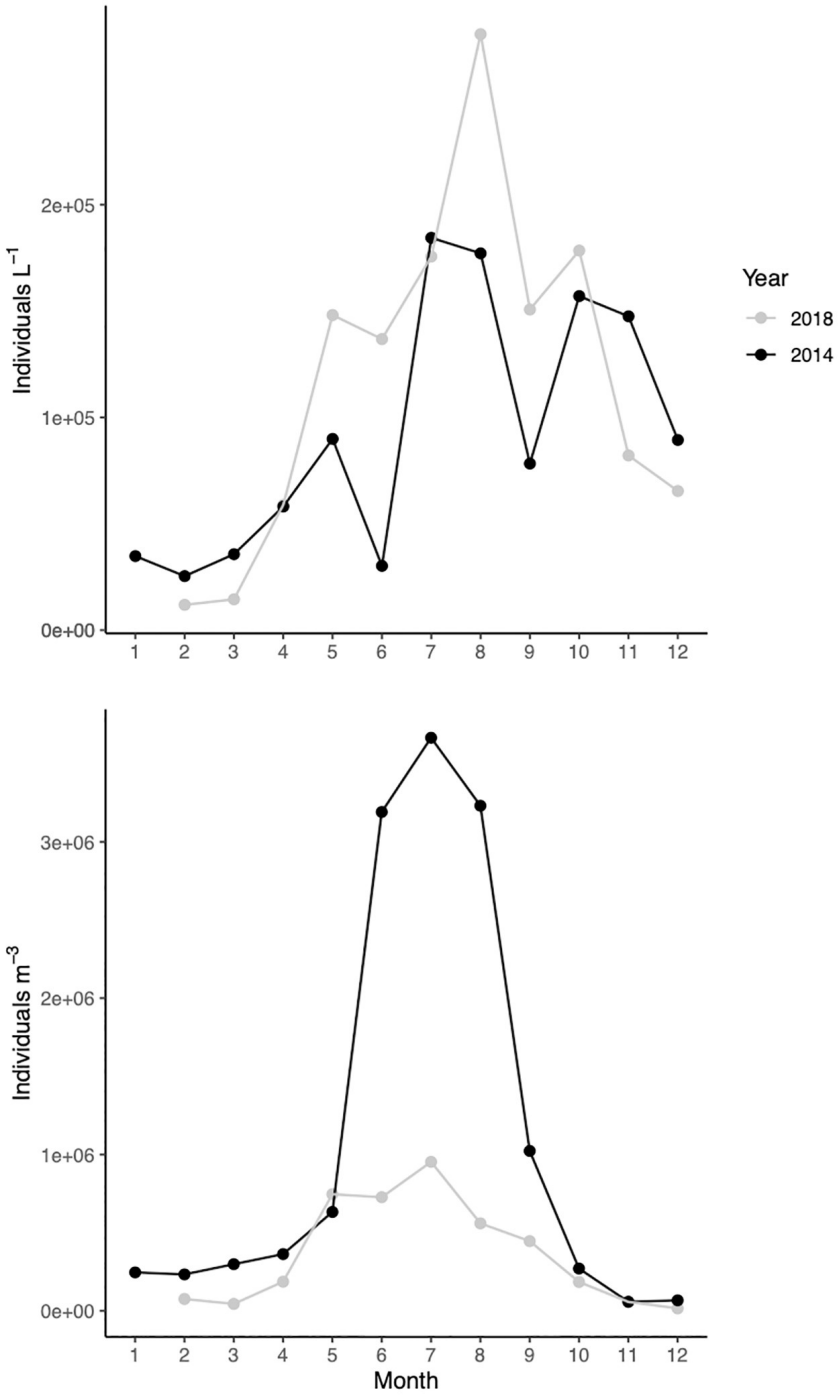
Seasonal dynamics of total metazooplankton (lower) and ciliates (upper) in 2014 (gray line) and 2018 (black line).

In winter 2014 large herbivorous ciliate species were dominant (e.g. *Tintinnidium fluviatile*, *Limnostrombidium spp*., *Pelagostrombidium spp*., *Rimostrombidium spp*.) with relatively high abundances (~32 ind mL^-1^). The maximum ciliate numbers occurred in July (~184 ind mL^-1^, Fig 7, upper) and were dominated by small picovorous species from generas *Cyclidium*, *Uronema*, *Balanion*, *Rimostrombidium* and *Halteria*. In 2018 the smallest abundance of ciliates occurred in winter (~10–15 ind mL^-1^, Fig 7, upper), the most common genera being *Coleps*, *Urotricha* and *Mesodinium*. Highest abundances were recorded in August (~280 ind mL^-1^) dominated mostly by similar species as in 2014.

In 2014 metazooplankton biomass peaked in June and consisted mainly of cladocerans *Chydorus sphaericus* 42% and *Daphnia cucullata* 24%. Yet, in terms of abundance, rotifers (mainly *Anuraeopsis fissa*) were displaying very high numbers (exceeding 2800 ind L^-1^ Fig 7, lower) during June-August 2014. In 2018 overall abundance of metazoans was smaller than long-term average in Lake Võrtsjärv. The summer maxima was lower (953 ind L^-1^ Fig 7, lower) than expected as the abundance of rotifers *Anuraeopsis fissa* and *Keratella tecta* was considerably smaller than usual.

### Abiotic and biotic factors affecting picoplankton

The results of forward stepwise multiple linear regression models indicated that throughout both years PPP-Chl *a* in Lake Võrtsjärv was mainly realted to four abiotic variables—TP, orthophosphate (PO_4_-P), water temperature and Secchi depth. The most influential biotic variable was the abundance of metazooplankton that was inversely related to PPP-Chl *a*. These variables explained ~80% of total variation of PPP biomass (Table 2). However, Peuk-Chl *a* was less affected by nutrient-related variables and mainly related to water temperature, Secchi depth, pH, dissolved oxygen (DO) and the abundance of metazooplankton (explaining ~63% of variation). Picocyanobacteria biomass was associated to TP, PO_4_-P and the abundance of metazooplankton explaining ~68% of the variation.

**Table 2:** Results of multiple linear regression models for predicting the Chl *a* biomass of photoautotrophic picoplankton (PPP-Chl *a*), picoeukaryote Chl *a* (Peuk-Chl *a*) and picocyanobacteria Chl *a* (Pcy-Chl *a*) in Lake Võrtsjärv.

Variable	Coefficients			Model	
	Estimate	T	P	Adjusted R2	P
PPP-Chl *a*					
Intercept	-2.12	3		0.79	<0.01
PO_4_-P	-34.02	-2.934	<0.05		
Secchi depth	-2.808	1.143	<0.05		
TP	55.29	4.030	<0.05		
Water temperature	0.415	2.27	<0.05		
Metazoans abundance	-0.00281	-5.246	<0.001		
Peuk-Chl *a*					
Intercept	-10.72	-1.469		0.63	<0.05
Water temperature	-0.3242	-2.196	<0.05		
PH	5.921	2.431	<0.05		
Secchi depth	-1.532	-2.218	<0.05		
Metazoans abundance	-0.000708	-2.335	<0.05		
DO	-0.156	-2.673	<0.05		
PO_4_-P	-4.994	-0.947	0.36		
Pcy-Chl *a*					
Intercept	-0.4774	-0.143		0.68	<0.001
Secchi depth	-3.621	-2.852	<0.05		
TP	42.41	2.771	0.05		
PO_4_-P	-20.87	-1.615	0.121	0.126	
Metazoans abundance	-0.00168	-4.118	<0.001		

In 2018 a strong positive relationship (r_p_ = 0.66, p<0.05) was detected between ciliates and picocyanobacteria while very strong negative relationship (r_p_ = -0.81, p<0.01) occurred in 2014 between picocyanobacteria and metazooplankton abundance. Over the two years pico-size diatoms were positively (r_p_ = 0.56, p<0.01) correlated to ciliate biomass. In 2014 there was also a positive relationship with pico-size diatoms and metazooplankton abundance (r_p_ = 0.67, p<0.05).

## Discussion

Large surface area and shallowness of Lake Võrtsjärv makes the phytoplankton community strongly controlled by physical parameters [48]. Our study clearly demonstrates that picoplankton Chl *a* dynamics in this type of environment cannot be explained by only one or two key variables. A widely accepted model proposed by Bell and Kalff [12] states that the PPP biomass could be easily predicted from total phytoplankton biomass via linear relationship between the two while with increasing total phytoplankton biomass the contribution of the PPP is expected to decrease. This differs from the findings presented here—in 2018 there was a positive relationship between total Chl *a* and PPP-Chl *a* (Fig 3a) although in 2014 the contribution of PPP was decreasing at higher total Chl *a* (Fig 3b). These results corroborate those of Somogyi et al. [22] who found that the model by Bell and Kalff [12] was unable to describe the association between phytoplankton biomass and PPP in shallow turbid freshwater environments. Somogyi et al. [22] suggested a turbidity-related ecological switching point (at 50 mg L^-1^ of inorganic turbidity) above which the turbidity is expected to be the main factor behind PPP predominance irrespective of total phytoplankton biomass. Our study confirmed that turbidity was associated to PPP-Chl *a* throughout the years. Both eukaryotic and prokaryotic components of PPP were strongly related to Secchi depth (Table 2). While feasible generalizations are available about oligo-mesotrophic temperate lakes, Silvoso et al. [20] also pointed out that finding clear straightforward patterns in eutrophic, hypertrophic and turbid lakes to predict phytoplankton size structure is problematic and requires further investigation. Weisse [49] noted that picoplankton abundance and trophic status in shallow and small lakes does not follow the same patterns as in large ones.

Unfortunately, the biomass estimations for PPP in shallow eutrophic lakes are still scarce and usually not presented in the units of Chl *a*, making our absolute values difficult to compare with previous studies. However, the contribution of picophytoplankton to total phytoplankton is more comparable and the average values for annual PPP contribution found in Võrtsjärv were within the range of variability recorded for picoalgae in other shallow, turbid eutrophic lakes (e.g. 22, 34). Our study showed that the amount and contribution of PPP in large and shallow eutrophic temperate lakes can vary significantly throughout the years. In 2014 the PPP contribution was smallest (~2–3%) in summer months and highest relative contributions (~18–21%) were recorded in February and March. A similar dynamics was noted in Lake Chaohu and Lake Taihu by Li et al. [34]. On contrary, in 2018 minimum contributions (0.4–4.5%) occurred from March to April and the PPP Chl *a* contributed most to the total biomass in September and November. In November, the Chl *a* concentration measured from the 0.2 μm filters exceeded the amount of Chl *a* on GF/F filters. This suggests that using GF/F filters for total phytoplankton biomass can be somewhat misleading as a small segment of picoplankton smaller than 0.7 μm could pass through GF/F filters. Therefore, the high abundances of small celled picoplankton such as *Synechococcus* or *Cyanobium* could not be fully represented on the GF/F filters. Similar effect was noted in a study conducted in the Baltic Sea [30]. Although Somogyi et al. [22] also found very high PPP contributions in turbid lakes, the extremely high PPP Chl *a* in autumn-winter 2018 in our study was a somewhat unexpected outcome that might have several explanations. The most obvious reason is related to the hydrological conditions deriving from the hot and dry spring and summer leading to extremely low water level towards the end of the year. These circumstances decreased the water transparency favouring shade-tolerant species like *L*. *planctonica* and *L*. *redekei*. Hence, the high quantity of picocyanobacteria revealed by Chemtax analysis during this period might also suggest a strong presence of low-light tolerant prokaryotes (e.g. phycocyanin-rich Pcy). Another underlying reason for the very high Pcy-Chl *a* concentration in this period might be related to methodology. Generally, large amounts of zeaxanthin would suggest high abundance of coccoid/pico-sized cyanobacteria [50, 51]. Yet, a note of caution is due here since, during the low-light periods, picocyanobacteria are likely to reduce the production of photoprotective pigment zeaxanthin that is used to identify cyanobacteria. Consequently, zeaxanthin: Chl *a* ratios will alter and the low ratio could potentially lead to some overestimation of cyanobacteria [52, 53]. Both Peuk and Pcy reached their maximum biomass in autumn. During this period, the water temperature had not dropped yet while the solar radiation was lower than in summer creating favourable conditions for both low-light tolerant picocyanobacteria and picoeukaryotes [54, 55].

### Taxonomic composition of PPP

Our study, aiming to determine the taxonomic composition of PPP on a seasonal scale in a turbid eutrophic system, highlighted cyanobacteria and diatoms as the major contributors to picoplankton biomass (Figs 4 and 5). According to the morphological studies, the most common pico-size genera for freshwater cyanobacteria are 1–2 μm long, 1 μm wide *Cyanobium* and 1–2 μm wide, 3–15 μm long *Synechococcus* followed by *Cyanothece* [56, 57]. The presence of several different strains of *Synechococcus* has been previously confirmed in Võrtsjärv and its inflows via molecular methods (pers. comm. Helen Tammert). Functionally also colonial picocyanobacteria should be included in PPP based on the size of individual cells, e.g. genera *Aphanocapsa*, *Aphanothece*, *Chroococcus*, *Coelosphaerium* [10, 57]. Ospina-Serna et al. [58] demonstrated that the presence of predatory cladocerans and rotifers increases the formation of colonies of individual cells. Although these colonial forms are present in Võrtsjärv, in this study they are only partly represented in PPP since the colony as a unit would not pass through the 2 μm filter. However, disaggregated colonies that break during the filtration can make their way through these filters. Still, their role in the microbial food webs is smaller compared to solitary Pcy that are essential food supply for nanoflagellates, ciliates and rotifers [10].

Regrettably, understanding about the taxonomic composition of picoeukaryotic algae in freshwater systems is still largely lacking although there have been some very good recent inputs about the community composition and diversity of Peuk [4, 24, 55, 59]. Diatoms are usually considered as a common part of microplankton (20–200 μm) but nearly 20 species of diatoms in marine systems have one of their cell dimensions below 3 μm [60]. The presence of diatoms in the PPP community has been noted in other eutrophic lakes usually involving small (2–3 μm) *Cyclotella* and *Stephanodiscus* cells. Earlier research has rarely found diatoms dominating among picoeukaryotes. However, more recent studies have started to broaden the picture about their occurrence. E.g. Li et al. [4], demonstrated using flow cytometry sorted samples with Miseq high-throughput sequencing that diatoms were an important group in eutrophic shallow lakes Taihu and Chaohu. Shi et al. [61] showed in a study done in more than 20 mesotrophic and eutrophic lakes that diatoms dominated the Peuk community in the most eutrophic ones. Shi et al. [59] found in highly eutrophic shallow Lake Chaohu that most abundant Peuk OTU in the lake was affiliated with *Stephanodiscus minutulus*. In Võrtsjärv both *Cyclotella* and *Stephanodiscus* are common diatoms that could have pico-size cells and very small *Stephanodiscus* cells have also been indentified via microscopy. Some small diatoms e.g. *S*. *minutulus*, can be very competitive in environments similar to Lake Võrtsjärv—highly fluctuating light in early spring or autumn, intense mixing and low temperatures [62]. It can thus be suggested that pico-size diatoms can be important players in eutrophic lakes.

Another issue that might contribute to the abundance of their marker pigment fucoxanthin in the pico-size fraction is the common occurrence of extremely thin and elongated diatoms e.g. *Synedra acus* (Ehrenberg), *Cylindrotheca closterium* (Ehrenberg) Reimann and Lewin in Võrtsjärv. Both might be able to pass the 2 μm prefilter due to their unique shape [32, 60]. Even very few of these large cells in pico fraction could significantly contribute to fucoxanthin concentrations.

The contribution of chrysophytes in the PPP community of Võrtsjärv appeared to be in range with previous studies by Li et al. [4], Shi et al., [24, 61] in other eutrophic lakes. The pigment analysis also suggested the existence of a small amount of pico-size dinoflagellates in Võrtsjärv. All known species of dinoflagellates exceed the size limit for picoplankton. Therefore, their appearance should be considered as a filtering artefact as some large flexible cells might be able to squeeze through small pores of the prefilters and accumulate on the 0.2 filters. This is a common problem in all studies that involve cascade filtration of the samples [23, 30, 63, 64]. Also, the debris of broken cells is able to pass the prefilters and can contribute to peridinin concentrations. Cell fragments of the larger cells are an unfortunate nuisance that cause problems also with flow cytometric analysis. Broken cells can be a source of misleading results that is rather difficult to avoid. Hence, the results of sequential filtering should always be interpreted with some caution as some cell fragments from larger fractions can still be present on the 0.2 μm filters. Nevertheless, sequential filtering is used and valued in studies of photoautotrophic picoplankton that requires catching the algal material on filters [29, 30, 63].

Additionally, it should also be considered that many organisms, including dinoflagellates and diatoms, may exhibit differential sizes during their life cycle—e.g. gametes, spores, vegetative forms [64, 65].

Contrary to expectations, this study did not find a considerable amount of chlorophytes among Peuk. Regardless of chlorophytes being considered the most common picoeukaryotes in freshwater systems [3, 4, 55], their abundance in Võrtsjärv remained below 4% within the Peuk and stayed relatively constant throughout the study period. At the same time, larger chlorophyte species are quite common in Võrtsjärv. Marker pigments chlorophyll *b* and lutein suggest that the group is present in the Peuk community but in much smaller quantity than diatoms. We expect that increase in their quantity would be reflected in higher pigment concentrations on the 0.2 μm filters. Even so, in future studies Peuk community in Lake Võrtsjärv needs to be studied on molecular level to further explore both abundance and composition of pico-size chlorophytes.

Our results corroborate the findings of other recent studies that have demonstrated the considerable variability of Peuk community structure in different freshwater ecosystems and various trophic states [4, 24, 61, 66]. While molecular methods give the most complete information about picoeukaryote community composition they lack in providing quantitative data and there are still problems attributed to the selectivity of DNA amplification techniques [28]. Thus, there is a continuing need for combining, improving and simplifying different methods to detect and quantify picoplankton and especially picoeukaryotes. Pigment-based chemotaxonomy proved to be an efficient and cost-effective alternative for determining the composition and dynamics of PPP in lakes. This approach should be employed in future studies that aim to understand the role of PPP in the food webs both in the context of eutrophication and climate change in freshwater environments. To our knowledge, this study is the first to provide information about the taxonomic composition of Peuk on a seasonal scale in Baltic lakes.

### Effect of abiotic and biotic factors

In Võrtsjärv total PPP-Chl *a* displayed a positive relationship with temperature. While water temperature showed no statistically significant association to Pcy-Chl *a*, it was an important predictor inversely related to Peuk-Chl *a* (Table 2). Our results support the idea of Peuk prevailing in low-light conditions and low temperatures as their predominance in the PPP community was also tightly linked to the presence or absence of ice cover (Fig 6). In the beginning of 2018, low temperatures together with constant ice cover accompanied by thick snow resulted in the absence of cyanobacteria from the picoplankton community. This suggests that picocyanobacteria are not able to maintain their biomass at low temperatures. Our finding is consistent with that of Vörös et al. [18] who found that in Lake Balaton picoeukaryotes were also better competitors than picocyanobacteria in the periods of low light and temperature when nutrients were not utilized at full rate. Several other studies [55, 67] have also observed the predominance of Peuk during winter. Moreover, it has been demonstrated that temperature is the most important factor affecting the diversity of Peuk group [59]. Our study suggests that the lack of ice cover brought on by a warming climate would favour picocyanobacteria in Võrtsjärv. We did not find cyanobacteria domination among picoplankton only in summer, as was seen in some earlier studies [18, 67]. Pcy biomass in Võrtsjärv was still high during autumn, as evidenced also by other researchers suggesting that some ecotypes of picocyanobacteria are fit for colder periods [55].

Weisse [49] proposed a hypothesis that in shallow lakes factors other than macronutrients (N and P) could have strong effects on PPP. In our study TP and PO_4_-P were both related to PPP-Chl *a* (p<0.05). Pcy biomass increased together with TP concentrations. As the nutrient availability in temperate regions is highest during winter, the maximum nutrient concentrations do not translate to highest PPP-Chl *a*. The light and temperature would constrain PPP during these periods limiting the photosynthesis as shown before by Somogyi et al. [55]. The Pcy biomass in Lake Võrtsjärv increases hand-in-hand with TP until it becomes overly cold and dark.

Tan et al. [68], suggested that larger cyanobacteria (e.g. *Microcystis*. *aeruginosa*) have competitive advantage at higher nitrate concentrations and small-sized *Synechococcus* sp. that is able to uptake nitrate rapidly, would dominate at lowest N concentrations. Therefore, picocyanobacteria might be more abundant in low nitrogen habitats. However, our study did not detect an apparent association between the nitrogen-related variables and Pcy in the eutrophic Lake Võrtsjärv.

Instead of macronutrients other physical parameters (temp, DO, pH, Secchi depth) were the main drivers for Peuk biomass. Similar observation has been made by Li et al [34] in shallow eutrophic lakes. As temperature is suggested to be one of the key factors shaping the Peuk community composition it should be kept in mind that not all taxa nor species within this group act the same [59]. The diversity and abundance can have significant temporal variation that needs further more detailed investigation.

In Lake Võrtsjärv it must be considered that the water level has been shown to be the leading force controlling light climate as well as nutrient cycles in Võrtsjärv. When water level drops, the nutrient concentrations, turbidity and phytoplankton biomass in Võrtsjärv increase [69, 70]. In 2018 the water level decreased drastically during the year and all the above-mentioned variables were affected. This was the highest water level amplitude recorded in Võrtsjärv since regular monitoring started in 1923. Similar effect was not well pronounced in 2014 as the water level was constantly low throughout the year. It can thus be suggested that the PPP biomass and dynamics are very strongly influenced by the water level in the lake. Lower water level increases the concentration of PPP-Chl *a* and higher turbidity paired to it gives a competitive advantage both to low-light tolerant Peuk and picocyanobacteria.

Our models showed that additonally to abiotic parameters, the biotic interactions were very important for explaining the variability of PPP-Chl *a*. We saw that the abundance of metazooplankton was very significant for predicting both Peuk and Pcy-Chl *a*. Also, the abundance of ciliates was linked to Pcy-Chl *a* in 2018 and to pico-size diatoms throughout the study. The abundance of ciliates in Lake Võrtsjärv is higher than in many other eutrophic temperate lakes as their community is weakly suppressed by metazooplankton [71]. Zingel et al. [72] demonstrated experimentally that the main grazers of pico- and nanoplankton in the lake are in fact ciliates and their community in the lake is bottom-up controlled [71]. The abundance of ciliates in both years was higher than the long-term average in Võrtsjärv. We didn’t detect that the ciliate abundance or biomass was an important component of the multiple linear regression models but we witnessed significant relationships between ciliates and larger PPP groups. The strong positive relationship (r_p_ = 0.66, p<0.05) detected between ciliate abundance and picocyanobacteria Chl *a* in 2018 suggests that picocyanobacteria are an important food source for ciliates. As ciliates are reproducing rapidly, fast development of small algae and bacteria quickly increases their abundance in Lake Võrtsjärv [72]. It was somewhat surprising that very low Pcy-Chl *a* values were noted from June to August 2014 resulting in the fact that Peuk prevailed during the summer months (Fig 6). A very likely reason for their rapid decline was the extraordinarily high abundances of small bacterivorous rotifer *Anuraeopsis fissa* that occurred simultaneously. The grazing pressure was further increased by the summer peak of ciliates dominated by small picovorous species. This is also concurrent with the inverse relationship (r_p_ = -0.81, p<0.01) found between metazooplankton biomass and picocyanobacterial Chl *a* in year 2014. Similar phenomenon did not take place in 2018. Ciliates reached their summer peak in August and the amount of picocyanobacteria did not drop. However, *A*. *fissa* that is usually present with high numbers at this time was showing much smaller abundances than normally in Lake Võrtsjärv. During both years pico-size diatoms were positively (r_p_ = 0.56, p<0.01) correlated with ciliate biomass. The fact that the Chl *a* of pico-size diatoms and metazooplankton abundance (r_p_ = 0.67, p<0.05) in the lake were tightly linked in 2014 suggests that they are important food source for both ciliates and metazooplankton. No similar statistically significant relationships were detected for larger diatoms. Tõnno et al., [73] has also demonstrated using pigment analysis that small non-filamentous diatoms have an important place in the diet of small crustaceans in Lake Võrtsjärv. Therefore, the dynamics of picoplankton depends a lot on the community composition of both ciliates and metazooplankton and the interactions between the latter. Although substantial research has been carried out on autotrophic picoplankton in lakes, very few studies exist which incorporate the biotic interactions in their work. In research that aims to explain PPP abundances, ciliate and metazooplankton data should always be included.

## Conclusions

Our study demonstrated that phototrophic picoplankton constitutes an important part of the phytoplankton community in Lake Võrtsjärv. The eukaryotic part of PPP was dominated by diatoms followed by chrysophytes and other minor groups. Ice cover strongly suppressed the growth of picocyanobacteria. Total phosphorus, turbidity and metazooplankton abundance had a clear relationship with PPP Chl *a*. A natural progression of this work could be to further analyze the importance of PPP in the food webs as the decrease of ice cover brought on by warming climate will likely alter the taxonomic composition of phototrophic picoplankton. Since PPP has a strong effect on the stability of aquatic ecosystems and since raised temperatures will likely increase the proportion of small-sized phytoplankton, the pico-size algae and their dynamics require more attention in order to obtain a general understanding of how phytoplankton communities, trophic transfer and ultimately lake ecosystems will respond to climate warming.
